# Neural Oscillations and the Initiation of Voluntary Movement

**DOI:** 10.3389/fpsyg.2018.02509

**Published:** 2018-12-18

**Authors:** Samuel Armstrong, Martin V. Sale, Ross Cunnington

**Affiliations:** ^1^Queensland Brain Institute, The University of Queensland, Brisbane, QLD, Australia; ^2^School of Health and Rehabilitation Sciences, The University of Queensland, Brisbane, QLD, Australia; ^3^School of Psychology, The University of Queensland, Brisbane, QLD, Australia

**Keywords:** slow wave brain oscillations, voluntary movement, conscious experience, decision making, readiness potential, free will, transcranial alternating current stimulation

## Abstract

The brain processes involved in the planning and initiation of voluntary action are of great interest for understanding the relationship between conscious awareness of decisions and the neural control of movement. Voluntary motor behavior has generally been considered to occur when conscious decisions trigger movements. However, several studies now provide compelling evidence that brain states indicative of forthcoming movements take place before a person becomes aware of a conscious decision to act. While such studies have created much debate over the nature of ‘free will,’ at the very least they suggest that unconscious brain processes are predictive of forthcoming movements. Recent studies suggest that slow changes in neuroelectric potentials may play a role in the timing of movement onset by pushing brain activity above a threshold to trigger the initiation of action. Indeed, recent studies have shown relationships between the phase of low frequency oscillatory activity of the brain and the onset of voluntary action. Such studies, however, cannot determine whether this underlying neural activity plays a causal role in the initiation of movement or is only associated with the intentional behavior. Non-invasive transcranial alternating current brain stimulation can entrain neural activity at particular frequencies in order to assess whether underlying brain processes are causally related to associated behaviors. In this review, we examine the evidence for neural coding of action as well as the brain states prior to action initiation and discuss whether low frequency alternating current brain stimulation could influence the timing of a persons’ decision to act.

## Introduction

The mammalian brain evolved to support naturalistic behaviors that enable interactions with our environment by choosing and initiating movements (Tinbergen, 1951). The perceived experiences associated with these intentional, voluntary acts are fundamental components of human functioning. Yet despite much interest in the neural bases of conscious volition, *what* happens in the brain *when* we decide to act remains an unresolved scientific question. Human movements can be broadly divided into two types: stimulus-driven and self-paced. Movements that are performed in response to external signals such as sensory cues are said to be stimulus-driven (externally triggered), whereas movements performed at times freely chosen by one’s self are said to be self-paced or self-initiated (internally triggered). Thus, the factors priming our conscious decisions to perform day-to-day movements exist along a continuum between internally generated and externally generated triggers.

Our review is primarily concerned with activity that is detected over motor regions of the human brain that occurs during the last ∼2 s before the onset of action. As is consistent with much of the relevant literature (for examples see: Kornhuber and Deecke, 1965; Deecke et al., 1976; Libet et al., 1983; Cunnington et al., 2002, 2003, 2005; Shibasaki and Hallett, 2006; Schurger et al., 2012; Di Russo et al., 2017), terms such as voluntary movement, self-paced action, and self-initiated motor behavior are used interchangeably and refer to the specific context where a movement is performed in the absence of an external trigger. Of the studies reviewed, the voluntary movements that are most commonly used in experiments are simple, self-paced actions, such as single finger flexions or button-presses. Also, our use of the term ‘decision’ and the phrase ‘conscious decision’ exclusively refers to a person’s final commitment for executing a movement. While our main focus is on simple voluntary movements, there is some discussion involving complex voluntary movements as well as stimulus-driven movements, but the distinction is always made clear.

The neurophysiological approach for investigating voluntary movement involves measuring electric brain signals before action takes place. These signals are commonly observed over pre-motor brain regions, including the supplementary motor area (SMA) and anterior mid-cingulate cortex (aMCC; Ball et al., 1999; Cunnington et al., 2003, 2005; Nguyen et al., 2014; Di Russo et al., 2017), as well as frontal regions such as the dorsolateral prefrontal cortex (Jahanashahi et al., 1995). When voluntary movements are performed, the activity over these areas shows changes in two kinds of electric brain signals: slow cortical potentials and neural oscillations.

The (slow cortical) readiness potential (RP), originally termed *Bereitschaftspotential*, is observed over motor regions in the electroencephalogram (EEG) as slow-building negative activity preceding movement onset (Kornhuber and Deecke, 1965; Shibasaki and Hallett, 2006; Di Russo et al., 2017). The so-called *classical interpretation* of the pre-movement activity observed in the RP is that gradual increases in negative brain potential over motor regions reflect a specific and goal-directed process for preparing the initiation of upcoming voluntary acts (Kornhuber and Deecke, 1965, 1990). In contrast, recent studies propose that the pre-movement activity represented by the RP reflects ongoing changes in underlying brain oscillations that merely alter the probability that a voluntary movement will occur (Schurger et al., 2012; Jo et al., 2013; Schmidt et al., 2016).

With regard to ongoing changes in brain oscillations, the initiation and termination of voluntary movement is characterized by patterns of increasing and decreasing synchrony of neural firing over motor regions, which is observed in the EEG as changes in power across particular frequency bands (Pfurtscheller and Lopes Da Silva, 1999; Shibasaki and Hallett, 2006). The brain oscillations that are most studied in the motor domain occur in oscillatory activity in alpha (8–12 Hz) and beta (13–20 Hz) frequency bands, contributing to the so-called mu rhythm (10–20 Hz; Hari, 2006). More recently, evidence has indicated that lower-frequency brain oscillations (≤2 Hz) are related to the timing of voluntary movements in a phase specific way (Schmidt et al., 2016).

Measuring slow-cortical and oscillatory brain signals before the onset of voluntary action provides temporal indices for examining the conscious decision to act as well as the initiation of movement. However, imaging techniques such as EEG that are commonly used to study these brain signals are limited to inferring function by correlating the activity in the brain with the timing of voluntary motor behavior. Recently, the exogenous delivery of an alternating current via transcranial electrical stimulation has been shown to entrain endogenous oscillations as well as influence the timing of perception (Neuling et al., 2012; Helfrich et al., 2014).

The aim of our review is to evaluate the role of underlying brain processes in the neural control of movement and examine how these underlying processes are temporally related with the conscious experience of deciding and the initiation of voluntary action. We discuss both historical and current concepts surrounding the RP origin and function and consider evidence that goes beyond the suggestion that pre-movement brain activity reflects a specific and goal-directed mechanism for preparing voluntary actions. We examine the electrophysiology underlying movement-related slow cortical potentials, paying attention to emerging evidence on slow-wave oscillations in the timing of voluntary movement. In addition, we detail further findings that suggest ongoing generalized processing and changes in underlying rhythms and brain states may provide a better account of the pre-movement activity observed in the RP. Finally, we assess the potential for transcranial electric stimulation techniques to probe a causal relationship between the phase of low-frequency brain activity and the timing of voluntary movement.

## The Readiness Potential

The discovery of the RP in 1964 by Hans Helmut Kornhuber and Luder Deecke has shaped modern research on volition. In their experiment, Kornhuber and Deecke (1965) recorded EEG from the scalp over the human motor cortex and electromyogram (EMG) from the fingers of participants while they performed a voluntary finger movement task. The participants were instructed to make self-paced finger flexions at irregular intervals for periods of up to a few minutes at a time (Kornhuber and Deecke, 1965). The movements were required to be abrupt and direct and the participants were told to wait for at least 15 s, but no longer than 25 s, between their movements. After averaging across many EEG epochs that were locked to the onset of the finger flexions, the recorded brain activity was observed to slowly rise in negativity, beginning approximately 1.5 s before the physiological onset of action. These findings lead to what is commonly termed the *classical interpretation*, where pre-movement increases in brain activity are thought to reflect specific and goal-directed preparation of upcoming voluntary acts. In this context, preparation refers to a chain of neural events that gradually increases the excitability in the motor cortex until a movement takes place.

The RP is an event-related potential, which means the onset of brain activity is time-locked to a particular event. In relation to the RP this event involves an intended or actual voluntary movement. The process for extracting the event-related RP from the EEG recording involves time-locking the EEG signal to the onset of the movement event and segmenting the signal into epochs to create trials (e.g., from -2000 to 500 ms relative to the onset of movement; Nguyen et al., 2014). By averaging across many repetitions of these trials the slow-rising pattern observed in the RP will emerge from the data.

The RP can be separated into distinct early and late components (Shibasaki and Hallett, 2006). The early RP (RP1) displays a slow-rising negative slope that starts roughly 1 to 2 s before movement onset, and is generated by pre-motor regions of the brain, including the SMA, pre-SMA, and aMCC (Romo and Schultz, 1987; Gerloff et al., 1997; Cunnington et al., 2002, 2003; Fried et al., 2011; Nguyen et al., 2014). In particular, the pre-SMA exhibits greater activity and earlier onset prior to actions where the timing of movement initiation is more self-paced than stimulus-driven (Deiber et al., 1999).

The late RP (RP2) shows a steepening gradient beginning about 400 ms before movement, and is characterized by local, lateralized activity with maximum amplitude occurring over contralateral motor and premotor cortices (Deecke et al., 1976; Cunnington et al., 2005; Di Russo et al., 2017). The lateralization of RP2, referred to as the lateralized readiness potential (LRP), is used to measure the extent to which the contralateral motor cortex is more active than ipsilateral motor cortex, relative to the hemisphere of the motor effector (Eimer, 1998). In summary, the pattern of activity observed in the RP1 component of the signal appears to reflect pre-movement planning and preparation, whereas the RP2 reflects processes specific to the limb to be moved.

Since first being reported, the RP has been classically endorsed as a preparatory signal that leads to the production of voluntary movement (Kornhuber and Deecke, 1965, 1990; Deecke et al., 1969; Shibasaki and Hallett, 2006). This endorsement rests on the general assumption that gradual increases in the firing rate of neurons preceding an event reflect a mechanism in the brain that is responsible for generating that event. But how is this pre-movement activity related to one’s conscious awareness of experiencing the decision to initiate a movement at a freely determined point in time?

## The Libet Experiment

One of the most prominent experiments involving the RP was conducted in the early 1980s by Benjamin Libet, who investigated the timing of peoples’ conscious decisions for performing voluntary actions (Libet et al., 1983). Here, the RP was used as a temporal marker of pre-movement activity in order to compare the time at which participants reported that they first became aware of the conscious experience of deciding to initiate an action (Libet et al., 1983). Similar to Kornhuber and Deecke (1965); Libet et al. (1983) also recorded EEG from the surface of the scalp and EMG from the index finger, which participants used to perform the self-paced finger flexion movements. While EEG was being recorded, Libet et al. (1983) measured the time at which participants’ first became aware of the conscious decision or ‘urge’ to move. To index the time that participants’ experienced the ‘urge’ to move, Libet et al. (1983) used an oscilloscope on which a dot moved around a circle at a rate of roughly 2.5 s per rotation; similar to a rapidly moving dial on the face of an analog clock. During the task, participants were instructed to make abrupt and capricious actions at unspecified times and to monitor the clock dial. At times when participants first became aware of the conscious decision to move, they noted the position of the dot on the clock-face. During the few seconds after completing the movement, the participant was prompted to report where the dot was positioned on the face of the clock by stating the time that was indicated by the clock to the experimenter. For example, if the dot was positioned half-way between the locations of ‘3’ and ‘4’ on the clock then the participant would report the time as ‘3:30.’ The time of this conscious decision to act was termed w-time (*will* time) as to reflect the spontaneous, ‘free will’ nature of the self-paced finger movements performed in the task (Libet et al., 1983; Libet, 1999). The findings reported by Libet et al. (1983) showed that the slow buildup observed in the RP preceded w-time by approximately 500–800 ms. The interpretation here was that the onset of preparatory brain activity occurs *before* people become aware of the conscious decision to initiate a voluntary movement (Libet et al., 1983). Such an interpretation challenged traditional dualist views of mind-body causation, which suggest that conscious decisions occur as a consequence of voluntary action. The straightforward assumption here is that the conscious decision to act cannot occur after the action has been performed since the pre-movement buildup of activity that leads to the decision to initiate an action (the assumed cause) does not take place after the action has already been initiated (the assumed effect).

Since the publication of Libet et al.’s (1983) original experiment, the rotating clock paradigm has been used extensively for investigating the temporal relationship between the onset of the RP and w-time (Haggard and Eimer, 1999; Lau et al., 2004; Sirigu et al., 2004; Soon et al., 2008; Fried et al., 2011; Schurger et al., 2012; Douglas et al., 2015). A study by Sirigu et al. (2004) showed that patients with focal cerebellar or parietal lesions were not able to report the time that they experienced a conscious decision to perform an action. Lau et al. (2004) reported that when compared to subjectively judging the time of one’s actions, judging the time of one’s conscious decisions was associated with greater activity over the pre-SMA. In particular, work by Soon et al. (2008) used a modified variant of Libet et al.’s (1983) clock to show that ongoing changes in underlying brain states can be used to predict peoples’ freely determined decisions up to several seconds before the decision is made. For this experiment, participants observed a random sequence of visually presented letters while functional magnetic resonance imaging data were obtained. The participants were instructed to use their index finger to perform self-paced button pressing movements and to verbally report the letter that was displayed on the screen at the time when they first experienced the conscious decision to act. Incredibly, the results showed that neural activity in fronto-parietal regions could be decoded to predict participants’ forthcoming actions up to 10 s before w-time was reported (Soon et al., 2008). Work by Fried et al. (2011) also used Libet et al.’s (1983) clock task, but instead recorded electrocorticography to assess the conscious decision to act at the single neuron level in patients that were being monitored for epilepsy. The authors observed activity in the pre-SMA and SMA that showed a slow increase in the firing rate of neurons that reflected the RP pattern and occurred roughly 1.5 s prior to w-time. These findings are well supported by other studies that show increased activity over SMA regions prior to the onset of voluntary movement (Romo and Schultz, 1987; Gerloff et al., 1997; Cunnington et al., 2002, 2003; Nguyen et al., 2014). The outcomes discussed here validate the initial findings reported by Libet et al. (1983) and provide empirical support for the argument that the brain prepares in advance of the time when people become aware of the conscious experience of deciding.

One potential explanation of the temporal relationship between the RP and w-time is that the conscious decision to act may be related to a chain of neural events that are activated before the onset of action (Libet et al., 1983; Schurger et al., 2012). This approach suggests that the brain prepares for the initiation of an upcoming movement while concurrently making forward predictions about the immediate sensory and somatosensory consequences (e.g., Blakemore and Frith, 2003). It may be that one’s awareness of deciding to act could arise from these predictions. Haggard and Eimer (1999) used the Libet clock (Libet et al., 1983) to investigate the relationship between brain activity and participants’ perceived time of voluntary action onset or their perceived time of the conscious decision to initiate voluntary action. In this experiment, participants performed self-paced button-press actions for which the authors classified into groups by categorizing actions according to whether participants made early or late judgments of w-time. The findings indicated that late judgments of w-time were associated with a delayed onset of the LRP (Haggard and Eimer, 1999). Since the LRP arises before the onset of action, this suggests that the subjective judgment of time relative to the conscious decision to act may be influenced by changes in underlying brain states prior to when the decision is made and the movement is performed. While these outcomes further suggest that the brain prepares for movement ahead in time of the conscious decision to act, the extent to which preparatory activity reflects a temporally protracted brain process that is responsible for the precise timing of consciously decided actions remains debated.

Despite receiving considerable support, Libet et al.’s (1983) suggestion that the ‘urge’ to move might develop in the brain before the time that a person consciously experiences the feeling of deciding to act has not been without opposition (Dennett, 1984; Wegner, 2003, 2004; Klemm, 2010; see also Dennett, 2015). Indeed, our conscious perceptions are delayed in comparison to events happening in real time, so it therefore takes several hundred milliseconds for sensory stimuli to reach the CNS and be processed into perceptions (Eagleman et al., 2005; Hallett, 2016). Since participants in Libet clock (Libet et al., 1983) experiments perform movements at times when the decision to act arises, it is unclear whether self-paced actions provide an accurate representation of the brain processes involved in volition, or instead that self-paced actions manifest in response to an internal trigger or neural cue in a way that is similar to how external triggers are processed for stimulus-driven movements (Bennett and Hacker, 2003; Kotchoubey, 2012). Work by Deecke and Kornhuber (2003) considered the complexity of Libet et al.’s (1983) interpretation, instead suggesting a more straightforward explanation as a possible alternative. According to Deecke and Kornhuber (2003), the very start of Libet clock (Libet et al., 1983) experiments requires participants to make an initial decision that they will be performing movements in a periodic fashion. This initial decision makes it possible that the decision for each movement made during the task is performed subconsciously outside of a person’s awareness and that consciousness is activated approximately 200 ms prior to movement onset. This 200 ms window of conscious experience allows enough time for the participant to terminate the preparation of the upcoming action before it is initiated.

A recent meta-analysis by Saigle et al. (2018) assessed a sample of studies (*N* = 48) that had used the Libet clock task (Libet et al., 1983) to investigate conscious experience and voluntary movement. Nearly all the studies in the sample had reported the occurrence of brain activity prior to participants’ awareness of their decision to act. Saigle et al. (2018) found extensive variation in the sample, pointing to inconsistencies in the methods applied and the findings and interpretations reported. In particular, Saigle et al. (2018) revealed that there were conflicting results for studies that had compared the timing of the RP signal to subjective reports of the decision to act (e.g., Haggard and Eimer, 1999; Schlegel et al., 2013). In addition, there were some studies where conclusions about the RP were drawn from work that did not include a measure of the RP (e.g., Radder and Meynen, 2013). Inconsistencies such as these are problematic in terms of drawing valid conclusions about the relationship between conscious decisions and the neural control of movement. Furthermore, the precise temporal relationship between the RP1 and RP2 components and w-time remains largely unclear. At the very least, if the time that a person experiences the conscious decision to act occurs after the onset of the RP as first proposed by Libet et al. (1983), then the precise physiological purpose of the RP remains questionable.

## The Readiness Potential: Specific Preparation or General Processing?

### Specific Preparation

The classical interpretation that pre-movement activity reflects a specific, goal-directed process for preparing upcoming voluntary movements is supported by evidence that properties relating to the upcoming movement influence the development of the RP signal (Shibasaki and Hallett, 2006; Di Russo et al., 2017). At the time of publication, there were two major reviews that collectively spanned the five decades of research that has been conducted on the RP (Shibasaki and Hallett, 2006; Di Russo et al., 2017; see also Deecke and Kornhuber, 2003; Jahanshahi and Hallett, 2003; Lang, 2003). Our purpose here is not to provide a comprehensive evaluation of the information contained in these reviews, but rather to build-on a theme of studies that assists in examining the classical interpretation of the RP.

The review by Shibasaki and Hallett (2006) discussed the first 40 years of RP research, including generator sources and the temporal onset of pre-movement activity in the brain (Praamstra et al., 1996; Yazawa et al., 2000), as well as how different activation patterns observed in the RP and event-related desynchronization indicate differences in the underlying brain processes (Pfurtscheller and Aranibar, 1977; Pfurtscheller and Lopes Da Silva, 1999; Pfurtscheller and Neuper, 2003). The review also considered how the duration and amplitude of the RP can be altered by properties of upcoming voluntary movements, such as the perceived effort and force exerted in performing the movement (Slobounov et al., 2004) as well as the complexity of the movement (Benecke et al., 1985; Kitamura et al., 1993; see also Lang, 2003).

The influence of the complexity of upcoming voluntary movements on the onset and amplitude of the RP signal has been attributed to the involvement of the SMA, which retrieves the necessary information from memory in order to prepare a short sequence of voluntary movements for execution in the near future (Roland et al., 1980; Roland, 1984; see also Lang, 2003). Here, the SMA first specifies the motor effector to be used (e.g., index finger) as well as the type of movement to be performed (e.g., pressing buttons on a keyboard) and then uses this information to prepare the motor sequence that is to be executed (e.g., initiate movement with the index finger to press the buttons ‘x’ ‘y’ ‘z’ on the keyboard). In a study by Benecke et al. (1985), EEG and EMG were recorded while participants completed four different voluntary movement tasks: (1) simple isotonic elbow flexions, (2) simple isometric finger flexions, (3) simultaneously performed isotonic elbow extensions and isometric finger extensions, and (4) sequentially performed isotonic elbow extensions and isometric finger extensions. Pre-movement activity was measured over the SMA, with results showing that the amplitude of the RP was greater for movements performed during simultaneous and sequential conditions than during simple conditions. These findings were supported by Simonetta et al. (1991), where the amplitude as well as the duration of the RP was larger for complex, self-paced movements (finger flexion followed by separately switching a button on and off) than simple, self-paced movements (finger flexion only). The straightforward interpretation of these findings is that increases in the amplitude and duration of the RP are directly related to increases in the complexity of the voluntary movement.

In contrast to previous reports on movement complexity (Benecke et al., 1985; Simonetta et al., 1991), a study by Kitamura et al. (1993) recorded EEG and EMG from the finger while participants performed self-paced finger extensions of the middle and index fingers on the right hand. The results showed that the amplitude of the RP was greater during a single extension of the middle finger than a dual extension of the middle and index fingers, and that the difference in amplitude was observed over the central region contralateral to the motor effector. One possible interpretation of these results is that increased activation of the hand sensorimotor area contralateral to the single-finger movement may suggest the involvement of the primary motor cortex (M1; Shibasaki and Hallett, 2006), where the enhanced amplitude of the RP reflects an increase in the resources required to perform the more-precise single finger movement. Since there are fewer muscles activated when extending one finger, the attenuation in RP amplitude for dual finger extensions may be associated with a motor control process that occurs independently of muscle mass activation (Kitamura et al., 1993). Alternatively, the difference in RP amplitude may be from additional cortical activity produced by motor inhibition processes (Shibasaki and Hallett, 2006), such as actively inhibiting versus actively performing the extension of the second finger. Despite differences in the outcomes of these studies (Benecke et al., 1985; Simonetta et al., 1991; Kitamura et al., 1993), the interpretations show how changes in pre-movement activity can be explained by a specific and goal-directed process, where differences in the duration and amplitude of the RP are attributed to the demands of the motor system for preparing and executing different types of voluntary actions. However, a potential limitation of these interpretations is that the different manipulations of movement complexity involve variations in cognitive demands, which may additionally influence parameters of the RP.

Building on earlier reviews (Lang, 2003; Shibasaki and Hallett, 2006), Di Russo et al. (2017) evaluated how the RP is influenced by additional movement-related properties, such as: pantomime (Luppino and Rizzolatti, 2000), grasping (Bozzacchi et al., 2014), virtual action (Bozzacchi et al., 2012), action observation (Tunik et al., 2008), self-paced saccades (Richards, 2003), bimanual actions (Debaere et al., 2001), fatiguing actions (De Morree et al., 2012), emotion-inducing actions (Herwig et al., 2007), stimulus-driven movements (Cunnington et al., 1997), simple response motor task (Di Russo et al., 2005), and praxis actions (Wheaton et al., 2005a,b). One notable conclusion suggested by Di Russo et al. (2017) was that in addition to motor regions, prefrontal and parietal cortices also showed activation during the pre-movement period, particularly when the upcoming movement was associated with increased cognitive demands.

In an experiment by Wheaton et al. (2005a), EEG and EMG were recorded while participants performed two types of self-paced praxis actions: transitive pantomimes (tool use movements: hammer-use, scissors-use, and screwdriver-use) and intransitive gestures (communicative movement: wave goodbye, show ‘peace’ gesture, and show ‘ok’ gesture). Compared to previous findings on simple, self-paced movement sequences (Benecke et al., 1985; Simonetta et al., 1991), both the transitive and intransitive actions showed similar patterns of pre-movement activity, which were detected roughly 3 s before the physiological initiation of movement, preceding the onset of the RP by about 1 s (Wheaton et al., 2005a). This activity was observed to originate over the superior parietal lobe before moving toward the inferior parietal lobe and reaching the anterior sensorimotor regions approximately 2 s before action initiation, or about the same time that the RP starts. The early activation of the parietal cortex may be attributed to the increased demands of higher-level cognitive processing and visuo-motor transformation associated with performing complex, praxis actions (Anderson and Buneo, 2002; Di Russo et al., 2017), such as initially perceiving an object through to preparing the motor-sequence and initiating the movement to achieve the proposed goal.

To establish whether the early activation of the parietal cortex was specifically related to complex, praxis actions, Wheaton et al. (2005b) recorded EEG and EMG while participants performed either self-paced pantomimes of tool-use movements or simple, self-paced thumb abductions. Results of this study revealed significant differences in pre-movement activity that were observed from -3 to -2 before EMG onset, where the amplitude of activity over the parietal cortex was greater for tool-use movements than thumb abductions. These findings support existing evidence (Anderson and Buneo, 2002; Wheaton et al., 2005a), where the involvement of parietal regions is attributed to the increased cognitive demands for preparing self-paced complex or praxis movements.

With regard to the roles of prefrontal and parietal cortices in the preparation and execution of self-paced actions, Di Russo et al. (2017) suggested that pre-movement activity does not reflect a specific preparatory process, nor does it exclusively depend on kinematic properties of the upcoming action. The authors further concluded that the processes underlying classical accounts of movement preparation involve numerous cognitive elements, such as those associated with movement complexity (e.g., Wheaton et al., 2005a,b). In consideration of the classical interpretation, one possible suggestion may be that cognitive demands reflect the involvement of psychological processes that are a necessary condition for performing complex voluntary movements, such as increased attention or executive function. In this context, the brain activity associated with such psychological processes might be explained as being *specifically* related to the task of *directing* voluntary motor behavior toward achieving a *goal*. At the very least, this application of the classical interpretation may provide some explanation for the role of psychological processes and task-related activity in the preparation of complex voluntary actions. But given that much of the research into voluntary movement involves simple actions such as pressing a button, it is interesting to consider how the classical interpretation might explain the influence of cognitive demands and task-unrelated activity on the duration and amplitude parameters of the RP during simple, self-paced movements.

A number of experiments have demonstrated that processes such as attention and motivation contribute toward pre-movement activity and influence the RP signal prior to simple voluntary acts (Keller and Heckhausen, 1990; Baker et al., 2011; Alexander et al., 2016). Work by Baker et al. (2011) showed that the amplitude of the RP was significantly reduced for voluntary movements at times when cognitive demands were high. While recording EEG, participants performed a self-paced finger movement task that involved pressing buttons on a response pad while they concurrently performed an *n*-back task (see Kirchner, 1958). The *n*-back task required participants to visually attend to lists of letters that were presented in a random serial order. Cognitive demands were manipulated across high- and low-load conditions. For the high-load condition, participants responded by pressing a button when they recognized a letter as being the same as the one that was presented two letters back. In the low-load condition, responses were made only when participants recognized a letter in the list that had been pre-specified prior to commencing the trial. The attenuated amplitude observed in the RP during the high-load condition suggests that limiting cognitive capacity inhibits pre-movement readiness to the extent that directing increased resources toward working memory comes at a cost for the upcoming voluntary act. However, despite the deficits observed in the RP signal, the performance of voluntary movements was not affected. Indeed, if the RP reflected a specific, goal-directed process for preparing voluntary movement then it could be expected that the changes observed in the RP signal would be reflected by variations observed in the physiological performance of the movement. An experiment by Freude et al. (1988) reported effects that were similar to those observed by Baker et al. (2011), where participants concurrently performed a voluntary movement task and a basic arithmetic task while EEG was recorded. Cognitive load was manipulated in the arithmetic task by varying the number of seconds that arithmetic problems were presented across short, medium, and long presentation times. Here, the observed attenuation in pre-movement activity during the shortest presentation time also suggests that underlying brain processes involved in voluntary movement preparation and cognitive control compete for similar resources.

In a study by Keller and Heckhausen (1990), EEG was recorded while participants performed a mental counting task and EMG of the finger was used in real time to detect when participants moved. At times when EMG onset was detected, the participant was asked to report whether their movement was performed consciously or occurred unconsciously. Comparing this EEG data for unconscious movements with EEG data of the same participants performing self-initiated movements in a standard Libet clock task (Libet et al., 1983), the results showed that the onset times of the RP were the same for self-initiated and unconsciously performed movements, but the amplitude of the RP was greater during self-initiated movements (Keller and Heckhausen, 1990). The results again suggest that cognitive processes may contribute toward pre-movement brain activity in simple voluntary actions. The interpretation here is that the differences in RP amplitude observed prior to self-initiated and unconsciously performed movements is due to the increased resources required for the conscious experience of deciding to act. The decreased amplitude of the RP during unconsciously performed movements suggests that cognitive resources were directed elsewhere and that the level of brain activity was below the perceptual threshold required to consciously experience movement.

In a recent experiment by Rigoni et al. (2011), participants were primed with information that sought to undermine their beliefs in ‘free will’ prior to completing a variant of the Libet clock task (Libet et al., 1983; see also Banks and Isham, 2009; Rigoni et al., 2010). While these results showed that inducing disbelief in ‘free will’ does not influence w-time, compared to participants who did not receive the ‘free will’ information, the primed group exhibited a significant attenuation in the amplitude of the RP (Rigoni et al., 2011). Specifically, the amplitudes of RP1 were smaller for participants who expressed a greater degree of disbeleif in ‘free will.’ There were no differences for the RP2. These results suggest that subjective views on ‘free will’ can influence pre-movement activity related to the preparation and execution of simple voluntary movements. They also suggest that the effect of abstract belief systems on levels of motivation might be more fundamental than what would be reasonably expected. The multiple cognitive and motivational manipulations discussed here that alter the development of the RP are challenging for the classical view, particularly given that the observed variations in RP seem at least partially dependent on non-motor processes.

More recently, Alexander et al. (2016) developed an adapted version of the Libet clock paradigm (Libet et al., 1983) where rather than using a single rotating dot the authors instead used four letters that were evenly spaced and rotated around a circle. For this version of the task, Alexander et al. (2016) instructed the participants to choose one letter and to note the position of the chosen letter on the clock face at the time that they made their decision. During this task, some trials involved choosing a letter only and other trials involved choosing a letter while concurrently pressing a button. A comparison of the decisions with and without the button-press responses showed that the RP preceded both types of decisions and displayed the same pattern for both types of trials. Therefore, the presence of the RP in the absence of a voluntary movement suggests that pre-movement activity in the brain may represent an underlying process that reflects general anticipation as opposed to specific preparation.

For tasks involving stimulus-driven movements where the timing is driven by an external signal such as a cue, the anticipation of this cue is preceded by another event-related slow cortical potential that draws similarities with the RP called the contingent negative variation (CNV; Walter et al., 1964). The CNV is a measure of attention and temporal expectation (Pfurtscheller and Neuper, 2003). Similar to the CNV and the RP, the stimulus preceding negativity (SPN) is another event related potential that is related to properties reflecting the expectancy of outcomes of self-determined behaviors (Hirao et al., 2016). Both the CNV and SPN provide an index of preceding brain activity for measuring participant performance on various cognitive tasks in the absence of voluntary movement. Despite differences between the self-paced RP and stimulus-driven CNV and SPN, each of these measures appear to share common processes involving preparatory activity in the brain (Tecce, 1972; Brunia, 1988). When taken together, the similarities across these findings are difficult to explain in terms of the primary assumption underlying the classical interpretation, where the RP is argued to reflect a specific, goal-directed process for preparing upcoming voluntary movements. Alternatively, the similarities observed in these event related potentials may be explained by a general, decision-making process, where brain activity preceding an event, whether it be motor or cognitive or otherwise, reflects a buildup in anticipation of an upcoming decision.

### General Processing

Having previously discussed the classical interpretation, we now consider more recent evidence which instead claims that pre-movement activity is better explained by general processing in the brain. A number of studies argue that the RP can be explained by spontaneous fluctuations in brain activity, where ongoing increases and decreases in the firing rate of neurons bias the timing of a person’s conscious decision to perform a voluntary action, such as moving their finger to press a button on a keyboard (Schurger et al., 2012; Jo et al., 2013; Murakami et al., 2014; Schultze-Kraft et al., 2016). This bias is evidenced by a relationship where the likelihood that a person will initiate an action is higher at times when the excitability is greater over motor regions (Jo et al., 2013; see also Schmidt et al., 2016). Interestingly, a similar relationship has been observed between the timing of stimulus-driven movements and the state of neural excitability, where the speed of a person’s reactions is faster at times when the firing rate of neurons in motor areas is higher. Commonly referred to as the *decisional interpretation* (Schurger et al., 2012), supporting studies claim that the RP reflects an ongoing general process for preparing upcoming decisions rather than a specific motor process for preparing upcoming movements. The basic idea of the decisional interpretation is that the buildup observed in the RP prior to the onset of voluntary movement would be the same as the buildup observed prior to the onset of any kind of decision being made. Given the novelty of Schurger et al.’s (2012) work, we first explain the decisional interpretation by using their stochastic accumulator model before reviewing further studies on the decisional interpretation of voluntary movement.

Around the same time as the Libet experiment (Libet et al., 1983), Eccles (1985) reported a tendency for voluntary movement to be initiated during the excitatory phase of spontaneous activity. Building on these findings, Schurger et al. (2012) developed a stochastic accumulator model to simulate the conscious decision of *when* to initiate action during self-paced movement tasks. Similar to the electrophysiological fluctuations observed in EEG recordings, Schurger et al. (2012) used 1/f noise (pink noise; see also Schurger, 2018) to reflect the fluctuating signal, as well as a threshold to reflect the moment of action onset and a drift component to reflect the perceived imperative to act. The perceived imperative to act refers to the general expectation that a movement is to be performed at some time in the future. Such an expectation is implicit in the demand characteristics of self-paced movement tasks, where participants rarely wait more than ∼20 s to move. As time moves forward, the perceived imperative to act increases along a shallow gradient, decreasing the distance between the fluctuating neural activity and the crossing of the decision threshold, resulting in the fluctuations being slowly ‘pushed’ closer toward the decision threshold (Schurger et al., 2012; Schurger, 2018). At times when the imperative to move is weak, the likelihood of a decision being made is influenced by spontaneous fluctuations in brain activity crossing the decision threshold.

The ability of the stochastic accumulator model to simulate the RP phenomena observed in human EEG data of self-paced actions was supported by empirical results from a voluntary movement experiment (Schurger et al., 2012). Here, Schurger et al. (2012) continuously recorded EEG while participants performed a modified variant of the Libet clock task (Libet et al., 1983). The analyses of the simulated data from the accumulator model as well as the empirical data from the Libet clock experiment each revealed patterns that reflected the RP. Schurger et al. (2012) conducted a further experiment using a modified version of the Libet clock task, where in addition to self-paced finger movements the task also included stimulus-driven finger movements. For the stimulus-driven movement condition, a brief audible tone was played at random times to cue the participant to respond by pressing a button as quickly as possible. The simulated data and the empirical data both showed patterns that reflected the RP, but when random interruptions were introduced to the simulated task, the model was fastest and slowest at responding when the preceding activity was nearest and farthest from the decision threshold, respectively. The simulation data involving the random interruptions was supported by the empirical data from the second experiment for stimulus-driven movements, where the randomly distributed auditory cues presented during a Libet clock task (Libet et al., 1983) cued participants’ reactions for pressing a button.

The stochastic accumulator model is largely based on the principles of evidence accumulation models (Schurger et al., 2012). Evidence-accumulation models have commonly been applied to perceptual decision-making (Linkenkaer-Hansen et al., 2004; Boly et al., 2007; Hesselmann et al., 2008; Mathewson et al., 2009). These models normally involve ongoing temporal integration of sensory evidence with internal noise until a threshold is reached, at which point a decision is made. However, in terms of voluntary actions, there is no sensory evidence to accumulate. Therefore, when movements are self-paced instead of stimulus-driven, the processes of evidence-accumulation and threshold-crossing, otherwise known as integration-to-bound, are governed by ongoing fluctuations in brain activity and the perceived imperative to act (Schurger et al., 2012). Importantly, while the ongoing changes in brain activity and the imperative to initiate a movement escalate the probability of self-paced action occurring, the neural fluctuations preceding movement are not as constrained by distinct onset and amplitude parameters such as those explained by the classical interpretation of the event-related RP. With regard to the timing of action, Schurger et al. (2012) argue that the decision to move occurs roughly ∼150 ms before the onset of action. Near to this time, participants report first becoming aware of the conscious decision to initiate a movement (Libet et al., 1983; Fried et al., 2011), and the activity observed during the LRP becomes lateralized (Haggard and Eimer, 1999). Accordingly, the RP1 and RP2 take place before and after ∼150 ms prior to action onset, respectively. In this context, the slow-rising pattern observed in RP1 (≥150 ms before onset) emerges as a result of summing across many near-threshold neural fluctuations captured by the ‘flash-photograph’ of time-locked, event-related averaging. The RP2 (≤150 ms before onset) involves the functional process of enacting the decision and executing the movement (Schurger et al., 2012). Therefore, if the temporal profile of the RP1 is due to an averaging artifact, then the classical assumption that pre-movement brain activity reflects specific, goal-directed processing seems questionable.

The decisional interpretation has been further supported by studies successfully implementing Schurger et al.’s (2012) accumulator model. Murakami et al. (2014) investigated spontaneous, self-initiated actions in a task where rats ‘decided’ when to abort waiting for a delayed tone. Specifically, the rats waited for a first tone, and once the tone was played the rats continued waiting a variable amount of time for a second tone, after which the rat received a large reward. However, occasionally the rat would become impatient and leave the waiting zone before the second tone was played, in which case they received a small reward. These ‘impatient’ trials were equated to voluntary acts. Recording from the secondary motor cortex (similar to human premotor cortex), impatient trials showed a slow negative rise that reached a particular threshold just prior to the rat leaving the waiting zone. When simulating the data with the accumulator model, results showed an RP pattern that was similar to empirical data for both rats and humans. These findings suggest that accumulator models identify the decision to act as a threshold-crossing moment while explaining how antecedent subthreshold activity can mediate action in the absence of a decision. Work by Schultze-Kraft et al. (2016) used a brain-machine interface during a goal-directed and reward-based learning task, where participants attempted to perform self-paced movements without being detected by the computer. At times when the computer made predictions a light went red, and if participants still performed the movement then the computer scored a point. The data for predicted and aborted trials showed that movements could successfully be aborted up to 200 ms before onset. These findings are consistent with suggestions that the decision to act reflects a final commitment to execute a movement.

Building on the decisional interpretation, Schmidt et al. (2016) claim that the onset of voluntary movement is related to the phase of infraslow oscillations (≤0.2 Hz; Vanhatalo et al., 2004). The authors quantify their assumption using results from single-trial analysis, suggesting that activity does not need to cross a threshold for movement to take place (Jo et al., 2013). Specifically, self-paced action was more likely during particular periods of motor cortical excitability, with the analysis showing that ∼66% of actions occurred during the negative phase and ∼33% during the positive phase. Since movements were not evenly distributed, event-related averaging would produce a shallow gradient similar to RP1. Considering these circumstances, EEG epochs time-locked to action onset would also be phase-locked to infraslow oscillations. Similar to repeated averaging of near-threshold neural fluctuations (Schurger et al., 2012; Murakami et al., 2014; Schurger, 2018), phase-locking could also account for the negative amplitude that emerges in the RP.

Taken together, these findings are difficult to explain using the classical interpretation for a number of reasons. The characteristic slow-rising pattern of the RP appears to be an artifact that is produced by event-related averaging of moment-to-moment fluctuations or infraslow oscillations that preceded action onset by no less than 200 ms (Schurger et al., 2012; Jo et al., 2013; Schmidt et al., 2016). In fact, Jo et al. (2013) reported that self-paced actions also occurred in the absence of rising negativity. Accordingly, the precise moment of the decision to act might not be exclusively primed by a specific, goal-directed process. Instead, the likelihood of movement taking place might be related to ongoing changes in oscillating brain activity, and to a lesser extent, the implicit task demands surrounding the perceived imperative to act. Therefore, emerging evidence suggests that activity occurring prior to voluntary movement reflects ongoing changes in general processing, instead of a specific neural event. Next, we discuss the role of neural oscillations in voluntary movement, with a specific focus on lower frequency rhythms and the influence of phase.

## Neuronal Oscillations and Voluntary Movement

Brain oscillations represent a ubiquitous organizational pattern of cortical network dynamics, which reflect emergent properties of multiple neural processes and are observed across five distinct frequency bands: Delta (0.5–4 Hz), theta (4–8 Hz), alpha (8–12 Hz), beta (13–20 Hz), and gamma (20–100 Hz). It has also been suggested that brain oscillations exist beyond the conventional EEG spectra (0.5 ≥ *f* ≤ 100 Hz), ranging from 0.01 to 600 Hz (Buzsáki and Draguhn, 2004). In particular, the execution and termination of action is characterized by patterns of enhanced or attenuated amplitude observed in alpha, beta, and gamma frequency bands (Jurkiewicz et al., 2006; Cheyne et al., 2008; Saleh et al., 2010; see also Hari, 2006). Generally, the amplitude of alpha and beta rhythms attenuates just prior to movement execution and resynchronizes after termination, whereas gamma remains enhanced from execution to termination. In addition to measures of amplitude, spectral properties such as phase and the cross-frequency coupling of phase and amplitude may also play a role in the timing of movements.

### The Phase of Neural Oscillations

The phase of neural oscillations carries temporal information that is distributed both within (Lakatos et al., 2008) and between brain regions (Varela et al., 2001). This rhythmic activity represents ongoing changes between states of high and low cortical excitability (Engel et al., 2001; Lakatos et al., 2007). Thus, depending on the phase of a particular brain state, identical exogenous events can lead to differences in perceived experiences. The cross-frequency coupling of phase and amplitude involves a relationship between the phase of lower-frequency oscillations and the amplitude of higher-frequency oscillations (He et al., 2010). This phase and amplitude coupling has been observed in different cortical regions (Freeman et al., 2003; Palva et al., 2005; Canolty et al., 2006; Isler et al., 2008; Sauseng et al., 2008), across the conventional EEG spectra (0.5 ≥ *f* ≤ 100 Hz); Lakatos et al., 2005), and extending into slower-frequency bands (≤1 Hz; Steriade et al., 1993a; Vanhatalo et al., 2004). Interestingly, phase and amplitude coupling exhibits characteristics of hierarchical organization (Lakatos et al., 2005). Speculating on earlier findings (see Schmidt et al., 2016), and considering the functional hierarchy of the motor system as well as models of voluntary movement (e.g., *what to move, when to act, whether to move at all*; Haggard, 2008), it is enticing to consider how coupling of gamma amplitude with beta phase and beta amplitude with alpha phase might flow-on to include lower-frequency rhythms such as delta or slow (Steriade et al., 1993a,b,c) oscillations.

The role of phase of brain oscillations has been extensively studied in perceptual and cognitive domains (Palva and Palva, 2007; Montemurro et al., 2008; VanRullen et al., 2011; Jensen et al., 2014; Bland et al., 2018). However, there are few studies investigating such phenomena in the motor domain, particularly in terms of lower-frequency oscillations and voluntary movement. As such, the spatial and temporal dynamics associated with the oscillatory phase of preparation and execution of self-paced action remains largely unknown.

### Slow-Wave Oscillations

We collectively refer to oscillations in infraslow (≤0.2 Hz; Vanhatalo et al., 2004), slow (0.1–1 Hz; Steriade et al., 1993a,b,c), and lower-delta (0.5–2 Hz) bands as slow-wave oscillations (SWOs; ≤2 Hz). SWOs reflect synchronized rhythms of neuronal membrane potential shifting between a hyperpolarized *down* state and a hypopolarized *up* state (Steriade et al., 1993a,b,c; Steriade, 1997). These slower rhythms have been generally associated with non-rapid eye movement (NREM) or *slow-wave* sleep, states of wakeful rest, and anesthesia (Steriade et al., 1993a). In addition, SWOs have been associated with maintaining an awareness of one’s self (Fransson, 2006), as well as one’s immediate environment and the passage of time (Fox and Raichle, 2007; Balduzzi et al., 2008). These findings indicate that SWOs are not limited to states where the brain is largely disconnected from the sensory environment, such as NREM sleep, and instead suggest that they may play a role in conscious processing.

In primary sensory cortices SWOs entrain to rhythmic stimuli, modulating the amplitude of higher frequency oscillations (Lakatos et al., 2007, 2008). Saleh et al. (2010) recorded local field potentials in M1 from a participant during a go/no-go task. A sequence of five spatial cues was presented and the participant selected either the second or the fourth cue depending on the trial. Findings showed that the phase of SWOs entrained to the inter-stimulus-interval (500 ms) and the amplitude of beta rhythms peaked momentarily before each cue up until the target (second or fourth cue). Here, the coupling of lower-delta phase with beta amplitude over M1 reflects periodic engagement with rhythmically presented cues. These findings suggest slow-wave phase reflects a temporal process that aligns higher frequency oscillations so they are optimally positioned for processing rhythmic information. Therefore, SWOs may act like an internal metronome for ordering the periodic enhancement of beta amplitude in preparation of expected stimuli.

Work by Kajihara et al. (2015) recorded EEG during a cued response motor task, where participants were free to choose either their left or right hand. Consistent with existing evidence, alpha rhythms attenuated over the motor region prior to movement execution (Pfurtscheller, 1992; Pfurtscheller and Neuper, 1992; Pfurtscheller and Lopes Da Silva, 1999; Pfurtscheller et al., 2006; van Wijk et al., 2012). In addition, coupling of delta phase with alpha amplitude displayed response-specific modulation over the motor cortex that was contralateral to the prepared hand (Kajihara et al., 2015). Specifically, the alpha power increased and decreased relative to the negative and positive phase of delta, respectively. Here, the phase of delta plays a temporal role that is independent of rhythmically presented cues, influencing the timing of conscious decisions. Though still behaving like an internal metronome, the absence of an external signal for which delta could entrain suggests, at the very least, that delta rhythms reflect either an underlying timing signal, or instead delta is driven by some other endogenous timing mechanism.

Recently, Hamel-Thibault et al. (2018) recorded EEG while participants performed speeded reaches toward visual cues using the hand of their choice. The findings revealed that delta rhythms were stronger over the hemisphere of the motor region contralateral to the selected hand, and that reach reaction times were lowest when the delta phase was closest to the negative peak. In addition, greater attenuation of beta band amplitude over both contra- and ipsilateral motor sites was correlated to faster reach reaction times irrespective of hand selection. Since lateralization of beta desynchronization occurs just prior to movement onset (Pfurtscheller et al., 2006; van Wijk et al., 2012), higher beta power at times when reactions were slower may suggest that the motor system was not sufficiently prepared for a hand movement to be selected and executed. Therefore, SWOs may have a temporal influence on the motor system such that the coupling of the phase of beta oscillations with the amplitude of delta oscillations biases the speed at which decisions can be made and movements can be initiated.

A comprehensive study by Combrisson et al. (2017) recorded stereotactic EEG during a four-alternative stimulus-driven movement task. Further to existing evidence of movement-related changes in alpha and beta bands (Pfurtscheller, 1992; van Wijk et al., 2012), phase and amplitude coupling was observed across delta, theta, and alpha bands over premotor regions specific to aMCC and SMA. These findings are consistent with evidence of aMCC and SMA involvement in the preparation and execution of both stimulus-driven and self-paced movements (Cunnington et al., 2002, 2003; Fried et al., 2011; Nguyen et al., 2014). In addition, single-trial analysis showed that the phase of slow-wave rhythms was the most significant factor in dissociating preparatory activity over pre-motor regions from executory activity over primary motor regions. Further to supporting a temporal role of SWOs in action timing and selection, these findings suggest that the phase transfers necessary information from a preparatory state to an actionable state.

Evidently, SWOs entrain to rhythmically presented visual stimuli (e.g., Saleh et al., 2010), however, in the absence of an external timing signal, SWOs exhibit an ongoing endogenous rhythm that operates like an internal metronome. This is highlighted by variations observed in reaction times, where the ongoing ‘up-and-down’ state that is synonymous with SWOs is said to influence performance (Garipelli et al., 2013). Furthermore, the presence of these ‘up-and-down’ states in stimulus-driven actions seems remarkably similar to the relationship between phase and self-paced actions observed by Schmidt et al. (2016; see also Jo et al., 2013). Although there is consistent evidence supporting the role of SWO phase on rhythmic entrainment, action selection, and response timing, these findings are largely limited to making inferences about stimulus-driven movements (Saleh et al., 2010; Kajihara et al., 2015; Combrisson et al., 2017; Hamel-Thibault et al., 2018). Despite including alternative-choice conditions, the constrained temporal parameters as well as the requirement for focal, sustained attention highlight the differences between stimulus-driven and self-paced tasks. However, the consistent movement-related patterns in beta and alpha bands, the activity observed over SMA and aMCC, as well as similarities with findings from recent decisional interpretation studies (Schurger et al., 2012; Schmidt et al., 2016) suggests that SWOs may also play a role in the timing of self-paced actions.

There is much value in replicating these studies to investigate the role of SWO phase in the timing of self-paced action (Saleh et al., 2010; Jo et al., 2013; Kajihara et al., 2015; Schmidt et al., 2016; Combrisson et al., 2017; Hamel-Thibault et al., 2018). However, due to the correlative nature of imaging techniques such as EEG, any such findings would be limited to making inferences only. In order to investigate further, one alternative could involve using transcranial electric brain stimulation to probe the causality of stimulus-driven and self-paced actions with the phase of SWOs.

## Transcranial Electric Brain Stimulation

Since the turn of the century there has been a rapid increase in the use of non-invasive brain stimulation to study cognition, relationships between brain and behavior, and the pathophysiology of neurologic and psychiatric disorders (Rossini et al., 2015). The transcranial electric stimulation technique involves the delivery of low-intensity electrical currents (∼1–2 mA) between at least two electrodes on the surface of the scalp for several minutes per session (∼5–30 min; Priori, 2003; Nitsche et al., 2008; Filmer et al., 2014). These currents generate an electrical field through the cortical tissue that modifies the resting membrane potential of neurons (Paulus, 2011). Transcranial alternating current stimulation (tACS) delivers an sinusoidal current at a set frequency, whereby the polarity of the current alternates between the anode and cathode (Neuling et al., 2012). These sinusoidal currents can modulate ongoing neural oscillations by artificially inducing up-and-down states similar to patterns exhibited by endogenous rhythms (Feurra et al., 2011; Helfrich et al., 2014). Building on relationships between brain oscillations and sensory processing and behavior (e.g., alpha oscillations and auditory perception, Rice and Hagstrom, 1989), recent studies have indicated that oscillations can be entrained by delivering tACS to the scalp at frequencies that are known to be related to particular sensory processes and behaviors (e.g., alpha frequency tACS and auditory processing, Neuling et al., 2012). In addition to providing support for existing correlational evidence, the major benefit of tACS studies is that they establish causal links between specific patterns of activity in the brain and changes in the associated behaviors (Helfrich et al., 2014; Cecere et al., 2015).

Many studies have used tACS at higher-frequencies (≥8 Hz) to further investigate observations in the EEG of movement-related synchronization and desynchronization of oscillations in alpha, beta, and gamma bands in motor regions of the brain (Antal et al., 2008; Pogosyan et al., 2009; Feurra et al., 2011; Schutter and Hortensius, 2011; Joundi et al., 2012; Wach et al., 2013; Cancelli et al., 2015; Pollok et al., 2015; Krause et al., 2016). However, few of these studies have investigated how the phase of such oscillations may influence the timing of movement onset. Converging evidence from animal studies shows that tACS entrains endogenous oscillations in such a way that the phase of stimulation modulates cortical excitability, leading to alterations in the associated behavior (Ozen et al., 2010; Ali et al., 2013). Such evidence indicates that applying a slow-wave sinusoidal current modulates the underlying brain rhythms and that the phase of the current synchronizes with the pattern of neuronal spiking. Indeed, these findings indicate that tACS modifies the timing of neural activity in a phase-specific manner.

In human studies, tACS entrainment of brain oscillations lead to phase specific modulations that had a causal influence on the perception of near-threshold sounds (Neuling et al., 2012) and visual targets (Helfrich et al., 2014). The phase of 6 Hz tACS over left frontal and parietal cortices influenced performance on a delayed letter discrimination task, where stimulating in-phase and out-of-phase lead to shorter and longer delays, respectively (Polanía et al., 2012). The tACS phase has also been shown to modulate physiological tremor in healthy participants (Mehta et al., 2014, 2015; Khatoun et al., 2018) as well as patients with Parkinson’s Disease (Brittain et al., 2013).

The duration of premovement activity shown by the RP is consistent with the duration of wave cycles in the frequency band of SWOs. Furthermore, evidence from recent EEG studies shows that the phase of SWOs over the motor cortex is related to initiation-times of self-paced action (Jo et al., 2013; Schmidt et al., 2016) as well as the speed of cued-responses for stimulus-driven action (Saleh et al., 2010; Kajihara et al., 2015; Combrisson et al., 2017; Hamel-Thibault et al., 2018). Therefore, considering that tACS is able to entrain neural oscillations at set frequencies and influence associated behaviors in a phase-specific way (Neuling et al., 2012; Brittain et al., 2013; Mehta et al., 2014, 2015; Khatoun et al., 2018), the delivery of sinusoidal currents at SWO frequencies may also influence the timing of people’s decisions to initiate action beyond their conscious awareness. An investigation of this type would provide further constraints for teasing apart the relationship between SWOs over motor regions, the role of cognitive processes in pre-movement activity, variations in the RP signal as well as differences for the RP1 and RP2 in the timing of the conscious decision to initiate voluntary movement.

## Summary and Future Directions

The conscious experience of deciding that is associated with the voluntary initiation of action is a key element of everyday life. A central objective in the study of voluntary action has been to identify how changes in neural activity are related to the timing of the conscious decision to act. Investigating this relationship has been particularly challenging since it is difficult to establish the causality of relatively unconstrained associations in a complex system such as the brain. These complexities are further highlighted by the varying contexts for which the decision to initiate movement can be primed, such as interactions between self-paced and stimulus-driven triggers, variations in the complexity of the movement and task-related psychological processes, as well as the involvement of non-motoric, cognitive and motivational processes. The challenge is made even more difficult by a lack of clarity on how to precisely measure the time these kinds of decisions take place. The aim of our review was to evaluate the role of underlying brain processes in the neural control of movement and examine how these underlying processes are temporally related to the conscious experience of deciding and the initiation of voluntary action.

The slow-rising pre-movement activity observed in the RP has long been endorsed as a specific and goal-directed preparatory signal that leads to the production of a voluntary movement (Kornhuber and Deecke, 1965; Shibasaki and Hallett, 2006). Approximately 20 years after being discovered, Libet et al. (1983) observed that the RP began roughly 800 ms before a person becomes aware of the conscious experience of deciding to act, otherwise known as w-time. One explanation of the temporal relationship between the RP and w-time is that the conscious decision to act may involve a chain of neural events that is activated during the few seconds before movement onset (Libet et al., 1983; Blakemore and Frith, 2003). Here, the brain prepares upcoming voluntary movements while also making predictions about the possible outcomes of the movement. However, it remains unclear if self-paced actions are the product of brain processes involved in conscious volition, or if instead self-paced actions manifest in response to an internal trigger in a way that is similar to performing stimulus-driven actions in response to an external trigger (Bennett and Hacker, 2003; Kotchoubey, 2012). Saigle et al. (2018) conducted a meta-analysis on studies that used the Libet clock paradigm (Libet et al., 1983). They reported extensive variations in the methods and findings of these studies, with conflicting results identified in studies comparing the RP signal and w-time as well as studies that had not measured the RP at all. Since it remains debated whether pre-movement brain activity reflects a temporal process for the conscious decision to act, and given the widespread variations in the methods and interpretations of the Libet clock (Libet et al., 1983), future studies might consider developing alternative paradigms for investigating ‘free will’ decisions and conscious volition.

The classical interpretation of the RP claims that the buildup of pre-movement activity reflects a specific and goal-directed process for preparing upcoming movements (Kornhuber and Deecke, 1965). This interpretation rests on the general assumption that gradual increases in the firing rate of neurons preceding an event reflect a mechanism that is responsible for generating that event. Furthermore, the classical view is supported by evidence that shows properties related to the upcoming action alter the onset and amplitude of the RP signal, such as the complexity of upcoming voluntary movements (Kitamura et al., 1993; Lang, 2003; Shibasaki and Hallett, 2006; Di Russo et al., 2017). However, studies that show how cognitive and motivational manipulations alter the development of the RP challenge the classical view, especially considering that such changes in the RP profile seem at least somewhat dependent on non-motor processes (Keller and Heckhausen, 1990; Baker et al., 2011; Alexander et al., 2016). Future studies looking to investigate the classical interpretation of the RP might also consider using new techniques or alternative experimental designs in order to examine the roles and contributions of underlying brain states in pre-movement activity.

The decisional interpretation of the RP suggests that the initiation of self-paced action is more likely to occur when slow fluctuations in neural activity are closer to a decision-threshold (Schurger et al., 2012). Given their focus on slow-changing brain activity, we propose that the findings reported by Schurger et al. (2012) and by Schmidt et al. (2016) reflect the same underlying process that is responsible for the timing of self-paced action. Taken together, the evidence suggests that oscillations in the infraslow band (0.01–0.2 Hz; Schurger et al., 2012; Jo et al., 2013; Schmidt et al., 2016) may be related to the timing of self-paced movements, such that the decision to act is more likely to occur during the negative phase than the positive phase of the underlying oscillations. Therefore, we contend that the early component of the event-related RP (RP1; ≥∼200 ms before action onset) might be attributed to repeated averaging of a disproportionate ratio (Jo et al., 2013) where more movements occur during the negative phase than the positive phase of SWOs. Importantly, this is not to dismiss existing RP studies, but rather to provide a platform from which to investigate previous findings in order to further establish how the underlying brain activity is related to the timing of voluntary movement.

Schurger et al. (2012) also found that response times of stimulus-driven actions were faster when slow fluctuations were nearer to the decision-threshold. Evidence suggests that SWOs, specifically oscillations in infraslow (0.01–0.2 Hz; Schmidt et al., 2016), slow (Combrisson et al., 2017), and lower-delta bands (Saleh et al., 2010; Kajihara et al., 2015; Hamel-Thibault et al., 2018) are related to the timing of stimulus-driven movements, such that the speed of reactions is more likely to be faster during the negative phase than the positive phase. Thus, there is much evidence to indicate that the temporal characteristics of voluntary movement may be governed by a mechanism that involves the negative phase of SWOs in motor regions of the brain. However, the collective shortcoming of these studies is that they are limited to inferring cause due to the correlational nature of imaging techniques, such as EEG. Therefore, delivering tACS at low frequencies (≤2 Hz) to entrain oscillations of the motor system may uncover a causal role for the phase of endogenous SWOs in the timing of voluntary movement.

## Author Contributions

SA, MS, and RC: conceptualization and structure. SA: writing–original draft. SA, MS, RC: writing–review and editing.

## Conflict of Interest Statement

The authors declare that the research was conducted in the absence of any commercial or financial relationships that could be construed as a potential conflict of interest.
